# Optimising trial monitoring on the AZURE trial

**DOI:** 10.1186/1745-6215-12-S1-A78

**Published:** 2011-12-13

**Authors:** Geraldine A Matthews, Roger Burkinshaw, Claire Davies, Vicky Hiley, Helen C Marshall, Robert E Coleman

**Affiliations:** 1 Clinical Trials Research Unit, University of Leeds, Leeds, UK; 2 Cancer Research Centre, Academic Unit of Clinical Oncology, Weston Park Hospital, Sheffield, UK

## Objectives

To assess telephone monitoring as a method of central monitoring for clinical trials.

## Methods

Determining how academic clinical trials units can optimise data quality via central monitoring methods is an important factor in trial management. In the AZURE trial, it became apparent from site monitoring that the endpoint dates as defined in EORTC literature [1] were misinterpreted at site. Although a training programme was initiated, some sites were seen to report date of confirmation rather than date of onset/suspicion as required. Therefore the focus of site monitoring was changed to review this critical endpoint data. Due to limited trial monitoring resources, a review of telephone monitoring was implemented.

A pilot phase was implemented to review the practicalities and possible results which could be obtained using telephone monitoring. During the pilot phase 17 trial endpoints were reviewed which had been verified by both site and telephone monitoring.

## Results

Agreement between site and telephone monitoring was achieved on 12/17 events during the pilot phase. Telephone monitoring was implemented and a total of 105 events were reviewed, some cases with multiple queries. Agreement between telephone monitoring and the Case Report Forms was found on 81/105 events. Discrepancies between the Case Report Form and telephone monitoring lead to a median amendment of the endpoint date of 9.0 days (IQR 7.0 - 23.0 days). In addition, 23 priority data queries were also identified for telephone monitoring which required clarification of diagnoses and addition of missing dates of suspicion with censoring of patients’ data if unresolved.

## Conclusions

Our findings indicate that although site monitoring remained the gold standard method of source data verification, telephone monitoring is a useful method for validating endpoint data when site monitoring resources are not available. The success of telephone monitoring was strongly related to the experience of the person at site involved with the review. When talking to less experienced staff, it was difficult to remotely navigate through the notes and pick up earlier scans/ recurrences. Also, if a participant had more than one recurrence it was found to be difficult to piece together all the available information over the phone.
